# Sinus venosus atrial septal defect presenting with brain abscess in a 33-year-old man

**DOI:** 10.15171/jcvtr.2019.53

**Published:** 2019-10-31

**Authors:** Roghayeh Pourkia, Seyed Habibollah Hassani, Simin Mouodi

**Affiliations:** ^1^Department of Cardiology, School of Medicine, Babol University of Medical Sciences, Babol, Iran; ^2^Department of Neurosurgery, School of Medicine, Babol University of Medical Sciences, Babol, Iran; ^3^Social Determinants of Health Research Center, Health Research Institute, Babol University of Medical Sciences, Babol, Iran

**Keywords:** Brain Abscess, Atrial Septal Defect, Transesophageal, Echocardiography

## Abstract

This study aimed to present a case of 33-year old man who was admitted with a history of one week headache and acute diplopia. No important finding was reported in his past medical history. Brain CT-scan revealed a large mass lesion in left parieto-occipital area with prominent vasogenic edema and midline shift. Brain magnetic resonance imaging (MRI) showed a mass with size of 5*4*5 centimeter with ring enhancement. After cranial surgery and removing the mass, transthoracic and transesophageal echocardiography (TEE) were conducted to find the source of brain abscess. Right ventricular (RV) and right atrial (RA) enlargement, significant left to right shunt, normal left ventricular (LV) and RV function, bidirectional shunt in addition to moderate size superior sinus venosus type atrial septal defect (ASD) were detected. Considering that most of brain abscesses have hematogenous source, a complete cardiac evaluation including TEE with contrast study is suggested for evaluation of patients with brain abscess.

## Introduction

Brain abscesses account for 8% of intracranial masses in developing countries; with a general incidence rate of four cases per million population, worldwide.^1^ They can result from pericranial contagious focus; direct inoculation or hematogenous seeding of the brain.^2, 3^ Hematogenous spread accounts for 9%-43% of brain abscesses.^4^ The pulmonary circulation acts as a filtering apparatus for systemic bacterial pathogens, therefore, in patients with right to left cardiac shunt bypass, hematogenous seeding to the brain is expected to occur more.^5^ Considering left-to-right shunts which are observed in atrial septal defects (ASDs), developing a brain abscess is uncommon in these patients.^6, 7^

Brain abscess presentation is variable and a high clinical suspicion is necessary for early diagnosis and prompt initiation of appropriate treatment.^8^ We present a case of cerebral abscess in an adult patient with left to right cardiac shunt due to ASD.

## Case Presentation

A 33-year old truck driver man was admitted to the hospital emergency department with a history of one week headache and acute diplopia. He stated that during recent week, he had a transient decreased level of consciousness resulting in a minor car accident. No important finding was reported in his past medical and drug history. At admission, his general appearance and consciousness level was normal; in addition to, vital signs including body temperature, blood pressure, pulse rate and respiratory rate were stable. No cervical stiﬀness or focal neurologic signs were found. Papillary edema was found in funduscopic examination.

The peripheral blood white cell count was 15000/μL with a neutrophil left shift (PMN = 80%). Blood culture was negative. There was no other positive laboratory finding. Chest X-ray and electrocardiogram (ECG) were normal.

**Figure 1:**
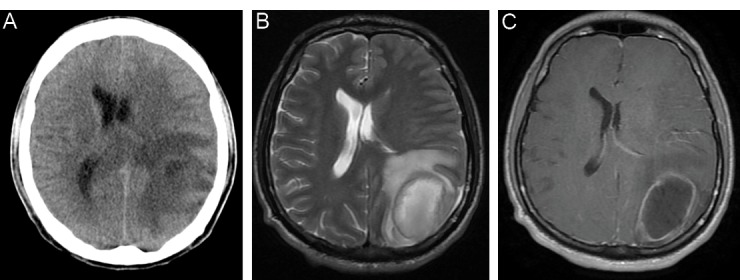
(A) Axial CT showing a mass with vasogenic edema in left parieto-occiput. (B) T2-weighted image showing the mass with a hypointense rim and surrounding edema. (C) Contrast-enhance T1-weighted image revealing the mass with well-defined peripheral rim enhancement.

The brain CT scan without contrast revealed a large mass lesion in left parieto-occipital area with prominent vasogenic edema and midline shift (Figure 1A). We requested magnetic resonance imaging (MRI) with and without contrast for more investigation (Figure 1B and 1C). There was a mass with size of 5*4*5 centimeter with ring enhancement in favor of a high grade glioma metastasis or brain abscess.

The patient underwent a left temporoparietal craniotomy. After opening of dura, we used transsulcal approach. During surgery, a purulent mass was observed. Complete drainage of purulent discharges and then irrigation was performed. Duraplasty was done to avoid potential complications of brain edema. Histopathology of biopsy specimen revealed a pyogenic abscess. Direct observation of purulent fluid under a microscope indicated a large amount of PMN cells. Furthermore, the cultures were negative.

To find the source of brain abscess, we requested CT-Scan of paranasal sinuses and mastoid part of temporal bone. There was no evidence of otitis media, paranasal sinusitis or mastoiditis.

Considering a hematogenous source, we did transthoracic echocardiography. It showed mild right ventricular (RV) and right atrial (RA) enlargement, significant left to right shunt (QP/QS=2), normal left ventricular (LV) and RV function with normal pulmonary artery pressure (PAP).

**Figure 2:**
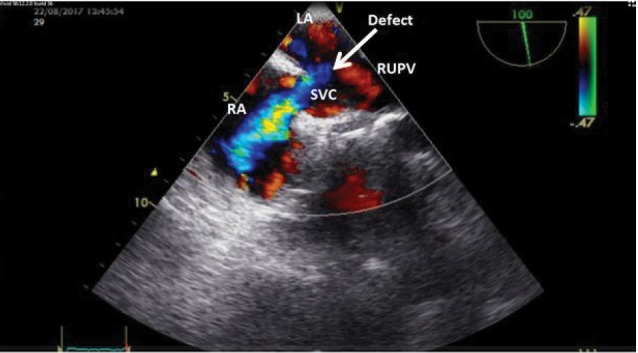
Transesophageal echocardiography showed sinus venosus type ASD with left to right shunt.

We requested transesophageal echocardiography (TEE) to determine the location of shunt and probable vegetation. Contrast study showed bidirectional shunt but predominant phase was left to right. In addition to, a moderate size superior sinus venosus type atrial septal defect (SVC type ASD) with partial anomalous right upper pulmonary vein connection was detected in TEE. There was no vegetation (Figure 2).

Patient received intravenous vancomycin, metronidazole and ceftriaxone for a total of 6 weeks and was cured. His headache was gradually resolved. At the time of discharge from the hospital, no lesion was detected in brain CT-scan. No physical and mental symptom was reported in 6 months follow up. In the control MRI which was provided 6 months after surgery no positive finding except mild arrested hydrocephaly was reported.

The patient did not accept our suggestion to undergo surgery for closure of ASD.

## Discussion

Brain abscess is rare in adults who have congenital heart diseases; and is often reported in cyanotic heart diseases such as tetralogy of Fallot in which right-to-left shunts occur.^9^

Sinus venosus ASDs account for 2%-10% of ASDs; this defect usually develops its symptoms as the patient ages.^6^ Some previous manuscripts presented cases of SVC type ASD presenting with brain abscesses in immunocompetent patients, similar to our study.^7^ Most of ASD patients have a left-to-right shunt,^10^ therefore, hematogenous abscess is not common in these patients.^11, 12^ In adults with ASD, pulmonary hypertension provides right-to-left shunt, cyanosis and paradoxical embolism. In ASD patients, echo contrast in left atrium can be representative of interatrial right-to-left shunt; which is observed with or without high pressure in the right side of heart. Shunt is a transient phenomenon which occurs when there is a pressure gradient on the sides of the atrial septum. At the beginning of systole, in brief times, the blood flows from the right to the left. This right-to-left shunt is limited and may easily be missed in acyanotic ASD patients, however, it can cause hematogenous brain abscess.^13^ In our study, despite normal pulmonary pressure, in contrast study, a right-to-left shunt was observed which can justify the paradoxical embolism and brain abscess in the patient.

This patient was 33 year old. A systematic review and meta-analysis revealed a male predominance of 2.4 to 1, and the mean age of brain abscess patients was reported 34 years.^14^ Some represented that brain abscess is more common in adult males younger than 30^1^; and others reported that most of brain abscess cases occur in the 3^rd^ to 5^th^ decades of life.^5^

This case manifested with prolonged headache and acute diplopia. In a previous study the most common clinical presentations of brain abscess were reported as headache (49%-93%), altered mental status (33%-70%), focal neurologic symptoms (29%-71%), fever (14%-88%) and nausea and vomiting (26%-71%).^5^ The patients´ symptoms are depended to the size and location of the mass lesions; and three symptoms including fever, headache, and the focal neurologic deficits can be observed in less than half of patients^1^; and the triad of fever, headache, and focal neurologic deficits could be present in 20% of these patients.^14^

It seems that in our patient the source of brain abscess was hematogenous. A research conducted on 52 brain abscess patients,^4^ and also a systematic review ^14^ showed that most common cause of brain abscess was hematogenous spread. A complete cardiac evaluation including TEE with contrast study is suggested for evaluation of patients with brain abscess.

This patient was treated with intravenous vancomycin, metronidazole and ceftriaxone; similar to our prescribed drug regimen, in a previous research a combination of cefotaxime, metronidazole with vancomycin was recommended as the empiric treatment of bacterial brain abscess.^15^

Considering that this patient refused to be treated with surgery, the recurrence of the disease is probable.

## Competing interests

None.

## Ethical approval

An informed consent was obtained form the patient regarding the publication of this report.

## Acknowledgments

Hereby, the cooperation of Clinical Research Development Center of Shahid Beheshti Hospital is greatly appreciated.
